# Comment on Manchaiah et al. Social Representations of “Tinnitus” and “Health” among Individuals with Tinnitus Seeking Online Psychological Interventions. *Audiol. Res.* 2023, *13*, 207–220

**DOI:** 10.3390/audiolres13040056

**Published:** 2023-08-14

**Authors:** Mirko Aldè, Giannicola Iannella, Jerome Rene Lechien, Francois Simon, Antonino Maniaci

**Affiliations:** 1 Department of Clinical Sciences and Community Health, University of Milan, 20122 Milan, Italy; mirko.alde@unimi.it; 2 Otology Study Group, The Young-Otolaryngologists of the International Federations of Oto-Rhino-Laryngological Societies (YO-IFOS), 75000 Paris, France; giannicola.iannella@uniroma1.it (G.I.); jerome.lechien@umons.ac.be (J.R.L.); f.simon@aphp.fr (F.S.); 3 Department of Human Anatomy and Experimental Oncology, Faculty of Medicine, UMONS Research Institute for Health Sciences and Technology, University of Mons (UMons), 7000 Mons, Belgium; 4 Department of Organi di Senso, University Sapienza, 00185 Rome, Italy; 5 Pediatric Otolaryngology—Head and Neck Surgery Department, Necker-Enfants Malades Hospital, 75000 Paris, France; 6 Faculty of Medicine and Surgery, “Kore” University of Enna, 94100 Enna, Italy

We read with pleasure the interesting paper titled “Social Representations of “Tinnitus” and “Health” among Individuals with Tinnitus Seeking Online Psychological Interventions” by Vinaya Manchaiah et al. [1]. The manuscript tackles a significant investigative query by delving into the societal perceptions of tinnitus and wellness among individuals pursuing internet-based psychological therapies.

The study used a qualitative approach, which is suitable for exploring the participants’ experiences and perspectives in-depth, and provided a detailed description of the methodology, enhancing the transparency and replicability.

However, we would like to share ideas on the publication. Firstly, the sample size is relatively small, which limits the generalizability of the findings. It would be beneficial to increase the sample size to enhance the robustness of the study’s findings and produce sharper results. Secondly, the study focuses only on individuals seeking online psychological interventions, which may not capture the perspectives of those who do not have access to or do not prefer online interventions. While this may not constitute a significant limitation, future studies could include individuals who seek face-to-face interventions or those who do not seek any interventions to provide a more comprehensive understanding of the social representations of tinnitus and health. Ultimately, a comprehensive examination of participants’ clinical features that could potentially influence tinnitus prognosis, such as hearing condition, hearing aid usage, history of ear and upper respiratory tract infections, previous surgeries and temporomandibular disorders, would be beneficial for future research [2,3,4,5].

Overall, the paper makes a significant contribution to the field of tinnitus research, and the findings have important implications for the development and delivery of online psychological interventions for a disease widely spread in the population and with significant impact on quality of life.
